# Biochemical Aspects of a Serine Protease from *Caesalpinia echinata* Lam. (Brazilwood) Seeds: A Potential Tool to Access the Mobilization of Seed Storage Proteins

**DOI:** 10.1100/2012/562715

**Published:** 2012-05-02

**Authors:** Priscila Praxedes-Garcia, Ilana Cruz-Silva, Andrezza Justino Gozzo, Viviane Abreu Nunes, Ricardo José Torquato, Aparecida Sadae Tanaka, Rita de Cássia Figueiredo-Ribeiro, Yamile Gonzalez Gonzalez, Mariana da Silva Araújo

**Affiliations:** ^1^Department of Biochemistry, Universidade Federal de São Paulo, 04044-020 São Paulo, SP, Brazil; ^2^School of Arts, Sciences, and Humanities, Universidade de São Paulo, 03828-000 São Paulo, SP, Brazil; ^3^Section of Plant Physiology and Biochemistry, Instituto de Botânica de São Paulo, 04045-972 São Paulo, SP, Brazil; ^4^Center of Protein Study, Facultad de Biología, Universidad de la Habana, 10400 Havana, Cuba

## Abstract

Several proteins have been isolated from seeds of leguminous, but this is the first report that a protease was obtained from seeds of *Caesalpinia echinata* Lam., a tree belonging to the Fabaceae family. This enzyme was purified to homogeneity by hydrophobic interaction and anion exchange chromatographies and gel filtration. This 61-kDa serine protease (CeSP) hydrolyses H-D-prolyl-L-phenylalanyl-L-arginine-*p*-nitroanilide (*K*_*m*_ 55.7 *μ*M) in an optimum pH of 7.1, and this activity is effectively retained until 50°C. CeSP remained stable in the presence of kosmotropic anions (PO_4_^3−^, SO_4_^2−^, and CH_3_COO^−^) or chaotropic cations (K^+^ and Na^+^). It is strongly inhibited by TLCK, a serine protease inhibitor, but not by E-64, EDTA or pepstatin A. The characteristics of the purified enzyme allowed us to classify it as a serine protease. The role of CeSP in the seeds cannot be assigned yet but is possible to infer that it is involved in the mobilization of seed storage proteins.

## 1. Introduction

Peptidases or proteases are essential molecules for the survival of all organisms as they can be involved in many aspects of development, physiology, defense, stress responses, and adaptation to the changes of environment. Also, proteolytic enzymes regulate protein processing and intracellular protein levels and remove either abnormal or damaged proteins from the cell, working as a cellular housekeeper [1].

Serine proteases are the best-characterized group of proteolytic enzymes in mammals and microorganisms. In recent years several of those enzymes have been isolated from various plant species, mainly from their seeds such as trypsin-like peptidases from *Glycine max* [2] and *Canavalia ensiformis* [3, 4], protease C1 from *Glycine max* [5], proteinase F from *Vigna radiata* [6], and subtilisin-like peptidases from *Glycine max* [7].

*Caesalpinia echinata *Lam. is a species of the Fabaceae (Leguminosae) family native in Brazilian forests that has been included in the list of species at risk of extinction [8]. Since 2000, our group has studied protein components from *C. echinata* seeds and we have already purified two serine protease inhibitors from them. *C. echinata* kallikrein inhibitor (CeKI) is able to inhibit human plasma kallikrein, plasmin, factor XIIa, factor Xa, porcine pancreatic trypsin [9], and human cathepsin G. *C. echinata* elastase inhibitor (CeEI) inhibits human neutrophil elastase, plasma kallikrein, and cathepsin G, porcine pancreatic elastase and bovine pancreatic chymotrypsin [10]. Inhibitory activity of these molecules upon proteolytic enzymes has been used to study and disclose molecular events involved in a lung edema model, paw edema, and inhibition of cell proliferation in a melanoma model (unpublished data).

It has been recognized that plant protease inhibitors belonging to Kunitz and Bowman-Birk trypsin inhibitor families [11] function as storage proteins in seeds. These proteins are essential for seed germination and normal plant development. The early initiation of storage protein mobilization occurs through the active proteases, such as cysteine, serine, aspartyl, or metallo proteases deposited, during seed maturation, in protein bodies and vacuoles. The start of storage protein mobilization indicates that the mechanisms protecting storage proteins against degradation during middle and late maturation have been overcome. However, only few experiments have been performed with isolated protein bodies to show if the incubation under appropriate conditions leads to the breakdown of internal proteins that could be attributed to the action of these proteases [12, 13]. Thus, we were interested to verify the existence of proteases from *C. echinata* seeds, similar to the ones described, which could be involved in mobilization of seed reserves of this leguminous during early growth. In the present work, we are reporting the purification and characterization of the first serine protease described in *C. echinata *seeds, named *C. echinata* serine protease (CeSP).

## 2. Material and Methods

### 2.1. Enzyme Purification

A crude enzyme preparation was obtained from *C. echinata *coatless dry seeds triturated and homogenized in 0.10 M Tris (purchased from Merck KGaA, Darmstadt, Germany) buffer pH 7.5 (1 : 20, w/v). The homogenate was centrifuged for 30 min at 2,300 g and the supernatant was subjected to an (NH_4_)_2_SO_4_ (purchased from Merck, Darmstadt, Germany) fractionation and allowed to stand overnight in cold. The resultant precipitated was recovered by centrifugation at the same conditions described above and dissolved in 50 mM Na_2_HPO_4_/NaH_2_PO_4_ (purchased from Merck KGaA, Darmstadt, Germany) buffer, pH 7.0. Proteolytic activity was examined in the hydrolysis of H-D-prolyl-L-phenylalanyl-L-arginine-p-nitroaniline (H-D-Pro-Phe-Arg-pNA) (purchased from Chromogenix, Italy) that was used to monitor the purification procedure. The sample with proteolytic activity was applied to a HiTrap Phenyl column (purchased from GE Healthcare Bio-Sciences AB, Piscataway, NJ, USA) equilibrated in 50 mM phosphate buffer, pH 7.0, 1.0 M (NH_4_)_2_SO_4_. Proteins were eluted in stepwise procedure with (NH_4_)_2_SO_4_ (0.95, 0.25, and 0.13 M) at a flow rate of 2.0 mL/min. The fractions with enzymatic activity were pooled, dialyzed against 20 mM Tris buffer, pH 7.5, and applied to a Resource Q anion exchange column (purchased from GE Healthcare Bio-Sciences AB, Piscataway, NJ, USA) equilibrated with 50 mM Tris buffer, pH 7.5. Proteins were eluted with a NaCl (purchased from Merck KGaA, Darmstadt, Germany) gradient (0 to 0.50 M) at a flow rate of 2.0 mL/min. The fractions containing enzymatic activity were pooled and submitted to a gel filtration on a Superdex 75 column (purchased from GE Healthcare Bio-Sciences AB, Piscataway, NJ, USA) equilibrated with 50 mM phosphate buffer, pH 7.0, 0.15 M NaCl, using blue dextran 2000 (2,000 kDa), BSA (67.0 kDa), ovalbumin (43.0 kDa), chymotrypsinogen A (25.0 kDa), and ribonuclease A (13.7 kDa) as standards (gel filtration calibration kit purchased from GE Healthcare Bio-Sciences AB, Piscataway, NJ, USA). Proteins were eluted in the same buffer at a flow rate of 0.66 mL/min, and the active fraction was stored at −20°C for further analysis.

### 2.2. Protein Quantification

Protein content was quantified according to the Bradford method [14] and using a standard curve of bovine serum albumin (BSA, purchased from Sigma-Aldrich Chemical Company Inc, Saint Louis, MO, USA).

### 2.3. Molecular Mass Determination

Sodium dodecyl sulfate polyacrylamide gel electrophoresis (SDS-PAGE) was performed on 12% slab gels according to Laemmli method [15]; the bands of reduced protein (50 *μ*g) were stained with Coomassie brilliant blue, and compared to the molecular mass markers from Kaleidoscope Prestained Standards (purchased from Bio-Rad Laboratories Hercules, CA, USA).

### 2.4. Optimum pH and Thermal Stability Determination

The optimum pH for the protease activity was determined in the H-D-Pro-Phe-Arg-pNA hydrolysis in a mixture of buffers (50 mM acetate/borate/phosphate—all components purchased from Merck KGaA, Darmstadt, Germany) to cover the pH range of 2.0 to 11.5. The enzyme (50 nM) was kept in each pH for 10 min at 37°C. After that, the substrate (0.40 mM) was added and the hydrolysis in each pH was followed by the absorbance at 405 nm (*ε*_pNA_ 8,990 M^−1^ cm^−1^ [16]) for 20 min in a Synergy HT microplate reader (purchased from Biotek Instruments, Winooski, VT, USA). For the thermal stability determination, the purified enzyme (50 nM) was first kept in 50 mM Tris buffer pH 7.1 for 30 min at different temperatures (20–60°C). Afterward, the temperature was kept at 37°C and H-D-Pro-Phe-Arg-pNA (0.40 mM) was added and the hydrolysis followed for 20 min at this temperature by the absorbance at 405 nm in the microplate reader.

### 2.5. Kinetic Characterization

Michaelis-Menten constants (*K*_*m*_) for the protease activity were obtained in the hydrolysis of H-D-Pro-Phe-Arg-pNA, H-D-valyl-L-leucyl-L-lysine-p-nitroanilide (H-D-Val-Leu-Lys-pNA), and N-benzoyl-L-isoleucyl-L-glutamyl (*γ*-OR)-glycyl-L-arginine-p-nitroanilide (Bz-Ile-Glu(*γ*-OR)-Gly-Arg-pNA) (all purchased from Chromogenix, Italy). The enzyme (50 nM) was kept in 50 mM Tris buffer, pH 7.1, for 2 min at 37°C. After that, a specific substrate was added in different concentrations (0.020 mM to 1.2 mM) and the hydrolysis of each substrate was followed by the absorbance at 405 nm, for 30 min at 37°C in the microplate reader. Two different experiments were performed in triplicate. The mean ± SD was obtained by statistical analysis using the commercial program GraphPad Prism version 5 (GraphPad Softaware Inc, San Diego, CA, USA). One-way analysis of variance with Bonferroni posttest was used.

### 2.6. Salt Influence on Catalytic Activity

The influence of different anions on the enzyme activity was determined using NaCl, NaBr, NaI, Na_2_SO_4_, NaH_2_PO_4_, and NaC_2_H_3_O_2_ (all purchased from Merck KGaA, Darmstadt, Germany) in the range of 0.50 to 1.0 M in 50 mM Tris buffer, pH 7.1. The enzymatic assays were performed as described above. The influence of different cations was studied using KCl, NaCl, NH_4_Cl, MgCl_2_, and CaCl_2_ (all purchased from Merck KGaA, Darmstadt, Germany) in the range of 0.10 to 0.70 M in the same conditions. 

### 2.7. Inhibition of the Enzyme Activity

The effect of inhibitors on the protease amydolytic activity was tested incubating the enzyme (50 nM) with irreversible inhibitors as, N^*α*^-tosyl-Phe chloromethyl ketone (TPCK—50.0 *μ*M [17]), purchased from Fluka BioChemika (Saint Louis, MO, USA), L-*trans*-epoxysuccinyl-leucylamido-butane (E-64—5.0 *μ*M [18]) purchased from Merck KGaA (Darmstadt, Germany), phenylmethylsulphonyl fluoride (PMSF—0.50 mM [19]), or N^*α*^-tosyl-Lys chloromethyl ketone (TLCK—50.0 *μ*M [17]), both purchased from Sigma-Aldrich Chemical Company Inc. (Saint Louis, MO, USA), in 50 mM Tris buffer pH 7.1, for 10 min at 37°C. The activity was also tested in the presence of reversible inhibitors as CeKI (21.5 and 215 nM [9]) purified by our group, benzamidine (0.50 and 4.0 mM [20]) purchased from Sigma-Aldrich Chemical Company Inc., (Saint Louis, MO, USA), pepstatin A (1 *μ*M [21]) purchased from USB (Cleveland, OH, USA), aprotinin (92 and 307 nM [22]), soybean trypsin inhibitor (SBTI: 49.8 and 498 nM [23]), or ethylenediamine-tetraacetic acid (EDTA: 1.0 and 10.0 mM [24]), all purchased from Calbiochem (Darmstadt, Germany), in 50 mM Tris buffer, pH 7.1, for 10 min at 37°C. After the preincubation, 0.40 mM H-D-Pro-Phe-Arg-pNA was added, and the substrate hydrolysis was followed for 30 min at 37°C at 405 nm in the microplate reader.

## 3. Results and Discussion

In the recent years, great efforts has been devoted to purification of plant proteases, which appear to be involved in several processes such as germination where specific degradation of cotyledon storage proteins occurs [25]. Also, they are thought to be useful toward understanding of several mechanisms at the subcellular level.

**Figure 1:**
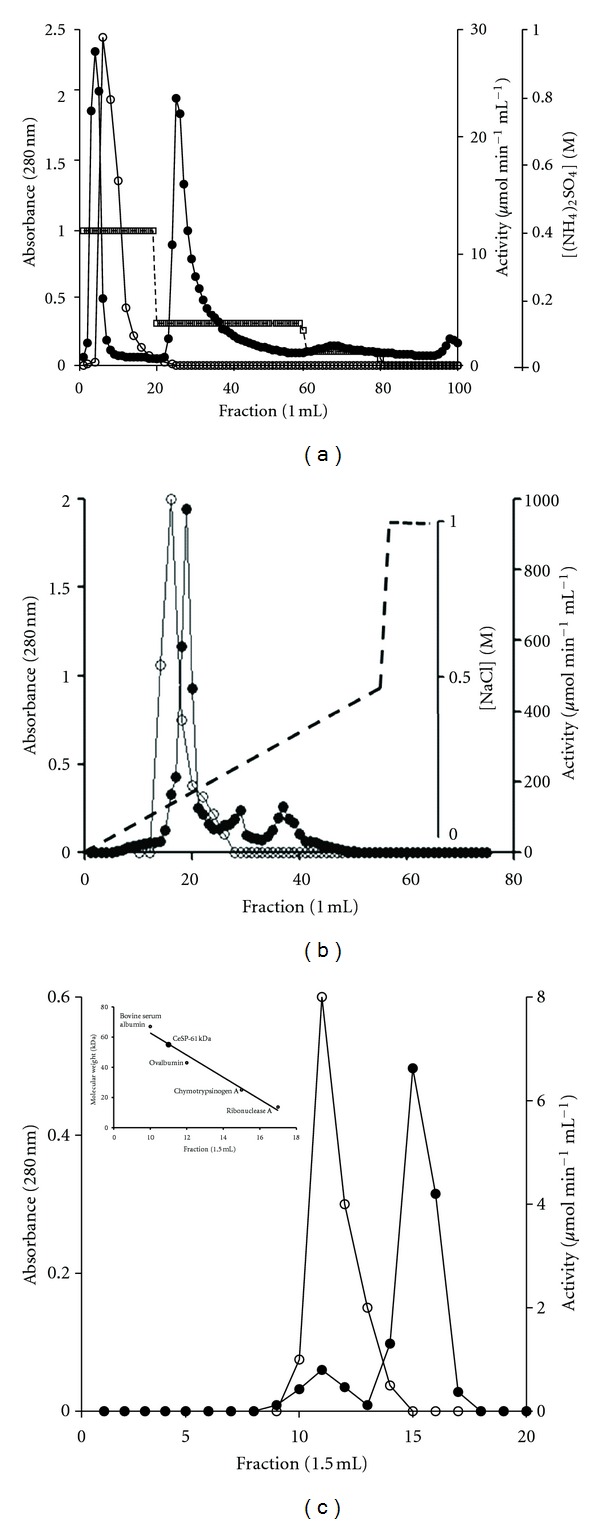
Elution profile of CeSP purification. (a) Hydrophobic interaction chromatography, where a Hitrap Phenyl column was equilibrated with 50 mM phosphate buffer, pH 7.0, 1.0 M (NH_4_)_2_SO_4_, and proteins were eluted with (NH_4_)_2_SO_4_ (0.95, 0.25, and 0.13 M). (b) Anion exchange chromatography, where a Resource Q column was equilibrated with 50 mM Tris buffer, pH 7.5, and proteins were eluted with a linear NaCl (0 to 0.50 M) gradient. (c) Gel filtration, where a Superdex 75 column was equilibrated with 50 mM phosphate buffer, pH 7.0, 0.15 M NaCl, and proteins were eluted in the same buffer. Inset: molecular masses as function of protein elution. Standard proteins: BSA (67.0 kDa), ovalbumin (43.0 kDa), chymotrypsinogen A (25.0 kDa) and ribonuclease A (13.7 kDa). All fractions were monitored by the absorbance at 280 nm (●) and enzymatic activity (°).

This work focuses on the isolation and characterization of a protease of *C. echinata* seeds—CeSP. The crude protease was obtained by precipitation on 40% (NH_4_)_2_SO_4_. Purification to homogeneity was achieved by hydrophobic interaction and anion exchange chromatographies (Figures 1(a) and 1(b)) and gel filtration (Figure 1(c)). The details of the purification analysis are summarized in Table 1. The specific activity of the purified enzyme was high (8,424 U/mg) as compared to the activity in the crude extract (176 U/mg). The total recovery of the activity was 8.2%. Besides, the enzyme was purified 47.8-fold.

**Figure 2:**
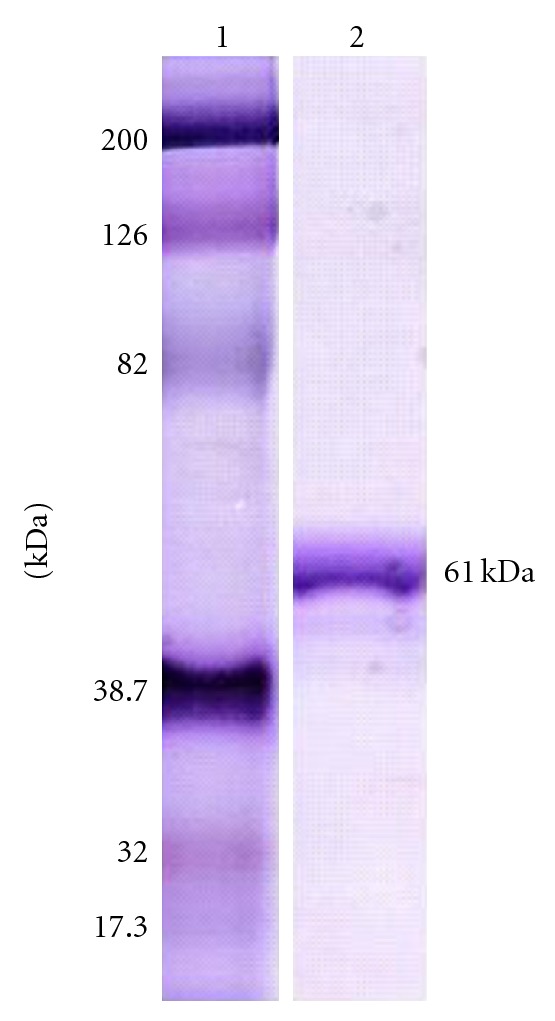
SDS-PAGE of CeSP. Gel 12%. Lane 1: molecular mass markers: myosin (200 kDa), *β*-galactosidase (126 kDa), bovine albumin (82.0 kDa), carbonic anhydrase (38.7 kDa), soybean trypsin inhibitor (32.0 kDa), and lysozyme (17.3 kDa). Lane 2: purified CeSP (50 *μ*g), under reducing conditions.

Although the molecular mass of plant serine proteases varies from 19 to 110 kDa, the majority lies between 60 and 80 kDa [26]. CeSP was showen to be a 61-kDa-protein, estimated in gel filtration on Superdex 75 (Figure 1(c) inset), and confirmed in SDS-PAGE (Figure 2). This value is similar to other serine proteases as dubiumin from *Solanum dubium* seeds (66 kDa) [27], serine endopeptidase from *Melothria japonica* fruits (61 kDa) [28], cucumisin from melon fruits (67 kDa) [29], trypsin-like protease from soybean seeds (59 kDa) [2], and two hydrolases from pea seeds (65 kDa) [30].

The enzyme activity was tested in the hydrolysis of some synthetic substrates. H-D-Pro-Phe-Arg-pNA, Bz-Ile-Glu(*γ*-OR)-Gly-Arg-pNA and H-D-Val-Leu-Lys-pNA were hydrolyzed by CeSP (Table 2). Z-Phe-Arg-pNA and Tosil-Gly-Pro-Arg-pNA were also hydrolyzed, but no N-Suc-Phe-pNA, N-Suc-Ala-Ala-Ala-pNA nor MeO-Suc-Ala-Ala-Pro-Val-pNA (data not shown). This fact shows the preference of the enzyme for basic amino acid residues (Arg or Lys) in P_1_ position, and aromatic residue (Phe) in P_2_ position. Similar results have already obtained [3, 4]. 

**Figure 3:**
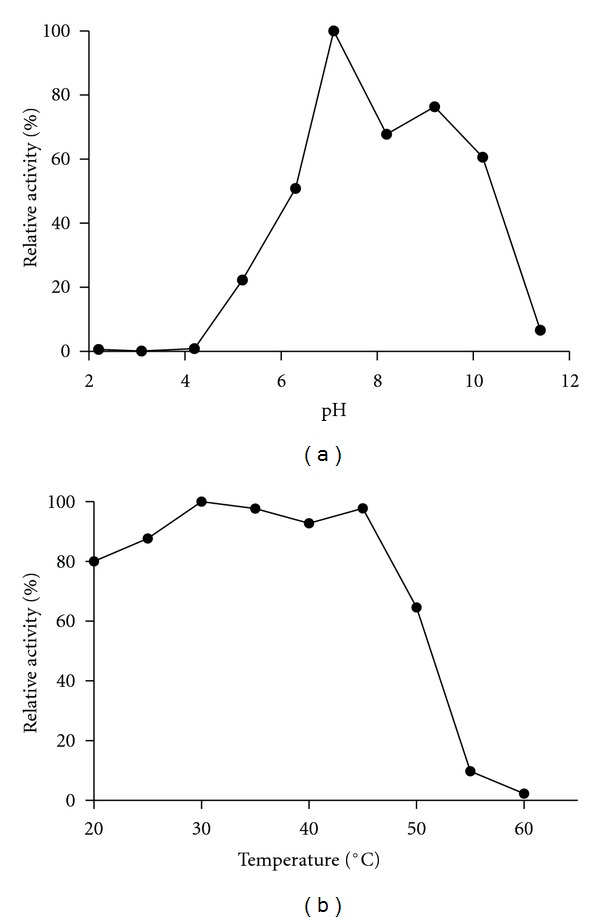
Effect of pH and temperature on the CeSP activity. (a) Optimum pH for the protease activity was determined in the H-D-Pro-Phe-Arg-pNA hydrolysis in 50 mM acetate/borate/phosphate buffer in the pH range of 2.0 to 11.5 for 20 min at 37°C. (b) For thermal stability studies, CeSP was kept in Tris buffer pH 7.1 for 30 min at different temperatures (20–60°C). Then, enzyme activity in the H-D-Pro-Phe-Arg-pNA hydrolysis was determined at 37°C in the same buffer.

About optimum pH and thermal stability, CeSP behaves as other described seed proteases. It exhibits a neutral optimum pH of 7.1 (Figure 3(a)) similar to a protease from soybean seeds, which works at pH 8.0 cleaving between two Arg residues, in the hydrolysis of *β*-conglycinin, a storage protein [31]. CeSP effectively retains its activity toward H-D-Pro-Phe-Arg-pNA hydrolysis at a temperature range of 20 to 50°C (Figure 3(b)), and this profile is similar to serine proteases from *Cucumis melo* [32], *Trichosants kirilowi* [33], *Hordeum vulgare* [34], and another leguminous seeds [3, 4].

**Figure 4:**
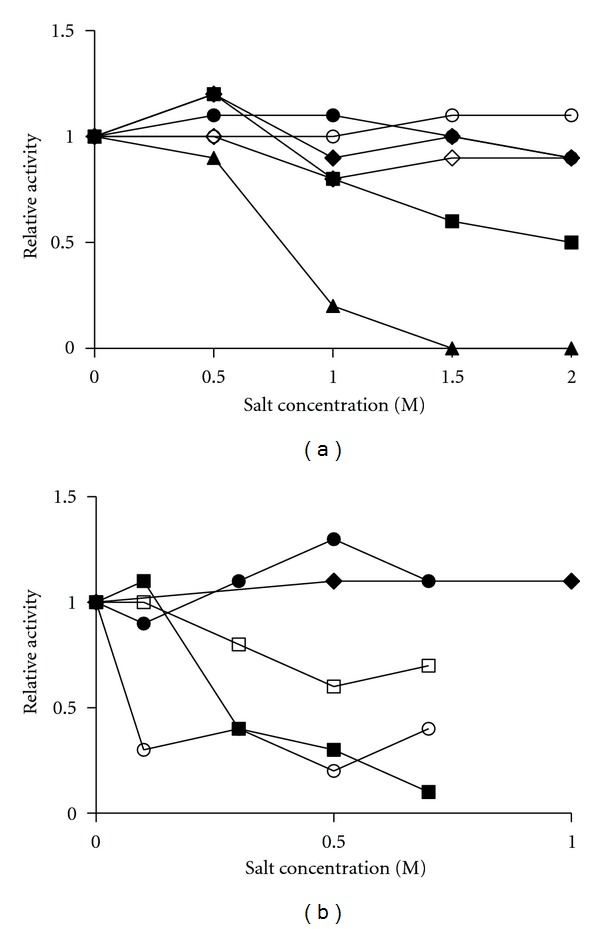
Salt influence on CeSP activity. Hydrolysis of H-D-Pro-Phe-Arg-pNA by CeSP was determined in absence or presence of (a) NaCl (●), NaBr (■), NaI (▲), Na_2_SO_4_ (*◊*), NaH_2_PO_4_ (°), and NaC_2_H_3_O_2_ (♦); (b) KCl (●), NaCl (♦), NH_4_Cl (°), MgCl_2_ (□), and CaCl_2_ (■).

The effect of ions on protein stability may be caused by chemical interactions between proteins and ions to form complexes. The ion specificity was mostly attributed to the ion ability to modify the water structure, thus influences the protein hydration environment. It has been realized that an optimal stabilization of enzymes could be achieved through the use of salts with kosmotropic anions and chaotropic cations [35]. Then, the effect of Hofmeister series on enzyme stability was determined in the enzyme activity in presence of the different salts. The enzyme remained stable in the presence of NaH_2_PO_4_, Na_2_SO_4_, NaC_2_H_3_O_2_, and NaCl, losing its activity in the presence of NaBr and NaI (Figure 4(a)). With the exception of chloride, the pattern of ionic interaction followed the Hofmeister series of salt hydration—PO_4_^−3^ > SO_4_^−3^ > CH_3_COO^−^ > Cl^−^ > Br^−^ > I^−^ [35]. The cations influence in the enzyme activity was also studied. CeSP remained stable in the presence of KCl, NaCl, and MgCl_2_, losing its activity in the presence of NH_4_Cl and CaCl_2_ (Figure 4(b)). With the exception of ammonium ion, the pattern of ionic interaction followed the Hofmeister series of salt hydration—NH_4_^+^ > K^+^ > Na^+^ > Mg^+2^ > Ca^+2^ [35].

In addition to the presented parameters, inhibition studies can provide a first insight into the nature of the enzyme, its cofactor requirement, and the nature of active site. As shown in Table 3, the strongest inhibition (about 96%) was observed with TLCK, an irreversible inhibitor of serine proteases. It is possible that alkylation of His residue in CeSP by TLCK leads to chemical modification resulting in the inactivation of the specific active site, which is responsible for the cleavage at positively charged residues at P_1_ positions of substrates, as reported by Imhoff and Keil-Dlouha [36]. CeSP was also inhibited by benzamidine (about 50%), an inhibitor of serine proteases, showing the CeSP affinity for Arg residues. So, these results may suggest that this protease is a serine protease.

As well as other serine proteases purified from leguminous seeds, *C. lineata* [4], *C. ensiformis* [3], and *G. max* [2], CeSP was not efficiently inhibited by protein inhibitors of serine proteases (aprotinin, SBTI, and CeKI), nor by PMSF (Table 3). Although these macromolecular inhibitors can affect activity of several serine proteases in animal physiological processes, they not necessarily inhibit correlated plant proteases. This result corroborates the fact that the proteins identified as protease inhibitors have others functions in seeds. Regarding to PMSF inability to block CeSP activity, it has been accepted that although serine proteases are structurally conserved enzymes, their catalytic triad may vary slightly and such a variation might account for the observed lack of inhibition of some serine proteases by PMSF.

To corroborate the hypothesis that CeSP is a serine protease, typical inhibitors of aspartyl, metallo, and cysteine protease were tested upon its activity. As shown in Table 3, these inhibitors had no effect on the enzyme activity, indicating that this enzyme did not belong to these classes of proteases. 

The physiological role of the CeSP was not elucidated yet and additional experiments need to be performed to address this question. However, several reports have documented the involvement of different proteases in the mobilization and cleavage of seed storage proteins upon seed imbibition and germination, which is an essential step in the establishment of the seedling [37]. The protein cleavage makes possible subsequent extensive breakdown catalyzed by other proteases such as endo- and exopeptidases. The proteolytic enzymes that degrade the storage proteins during mobilization identified so far are mostly cysteine proteases [11], but also include serine [6], aspartic, and metallo proteases [38]. However, mobilization of storage protein involves several members of a protease family or members of different protease families, the protease responsible for the initial degradation appears to be a serine protease [5]. This evidence indicates CeSP as a potential enzyme involved in the initial steps of mobilization of storage protein, which would contribute to understanding how plants switch from synthesis and accumulation of storage proteins during development to hydrolysis of those proteins upon germination.

Besides addressing elementary questions in plant protein metabolism, studies of the mobilization of seed storage proteins by proteases open the perspective to understand, for example, how these enzymes could be applied toward reduction of food allergens, many of which are storage proteins.

## 4. Conclusion

This is the first report of the purification and characterization of a protease from *Caesalpinia echinata *Lam. seeds. The molecular mass and enzymatic characteristics were allowed us to classify it as a serine protease. The role of CeSP in the seeds cannot be assigned yet but is possible to infer that it is involved in the mobilization of seed storage protein.

## Figures and Tables

**Table 1:** Typical steps of the CeSP purification.

Step	Protein^1^ (mg)	Total activity^2^ (U)	Specific activity (U/mg)	Yield^3^ (%)	Purification^4^ (fold)
Extraction	1,020	179,712	176	100	1
Fractionation	531	110,400	208	61.4	1.17
Hydrophobic interaction	68.0	20,064	295	11.2	1.67
Ion exchange	6.48	18,360	2,833	10.2	16.1
Gel filtration	1.75	14,742	8,424	8.2	47.8

^1^Total protein was estimated by the Bradford method. ^2^One unit (U) of enzymatic activity was defined as the activity that yields 1 *μ*mol of the product per minute under the conditions as described in Section 2.5.^3^Yield is given as percentage of isolated active material. ^4^Purification factor is calculated using the specific activity data.

**Table 2:** Kinetic parameters for hydrolysis of synthetic peptides by CeSP.

Substrate	*K*_*m*_ (*μ*M)	*k*_cat_ (s^−1^)	*k*_cat_/*K*_*m*_ (M^−1^ s^−1^)
H-D-Pro-Phe-Arg-pNA	55.7 ± 6.7	0.0053 ± 0.0002	95.2 ± 9.5
Bz-Ile-Glu(*γ*-OR)-Gly-Arg-pNA	297 ± 45	0.0091 ± 0.0006	30.6 ± 3.1
H-D-Val-Leu-Lys-pNA	2,09 ± 189	0.031 ± 0.002	14.8 ± 1.0

Hydrolysis of synthetic peptides (0.020 mM to 1.2 mM) by 50 nM CeSP in 50 mM Tris buffer pH 7.1, for 30 min at 37°C, was followed at 405 nm.

**Table 3:** Effects of some inhibitors on the CeSP activity.

Compound	Final concentration	Relative activity (%)
None	–	100
Serine protease inhibitors		
TLCK	50 *μ*M	4.30
Benzamidine	4.0 mM	57.4
PMSF	0.50 mM	69.2
SBTI	49.8 *μ*M	71.8
Aprotinin	307 nM	89.3
CeKI	215 nM	97.2
TPCK	50 *μ*M	119
Cystein protease inhibitor		
E-64	5.0 *μ*M	92.3
Aspartyl protease inhibitor		
Pepstatin A	1.0 *μ*M	77.6
Metallo protease inhibitor		
EDTA	1.0 mM	99.7

Irreversible (PMSF, TLCK, TPCK or E-64) or reversible inhibitors (aprotinin, benzamidine, CeKI, SBTI, EDTA or pepstatin A) were tested on the hydrolysis of H-D-Pro-Phe-Arg-pNA (0.2 mM) by CeSP (50 nM) in 50 mM Tris buffer, pH 7.1, for 30 min at 37°C. The release of p-nitroaniline was followed at 405 nm.
